# Toll-like Receptor 9 Induced Dendritic Cell Activation Promotes Anti-Myeloperoxidase Autoimmunity and Glomerulonephritis

**DOI:** 10.3390/ijms24021339

**Published:** 2023-01-10

**Authors:** Sharon L. Ford, Kim M. O’Sullivan, A. Richard Kitching, Stephen R. Holdsworth, Poh Yi Gan, Shaun A. Summers

**Affiliations:** 1 Centre for Inflammatory Diseases, Department of Medicine, Monash University, Clayton, VIC 3168, Australia; 2 Department of Nephrology, Monash Health, 246 Clayton Road, Clayton, VIC 3168, Australia; 3 Department of Pediatric Nephrology, Monash Health, 246 Clayton Road, Clayton, VIC 3168, Australia

**Keywords:** ANCA associated vasculitis, glomerulonephritis, TLR9, dendritic cell, myeloperoxidase, autoimmunity, kidney injury

## Abstract

ANCA-associated vasculitis (AAV) is intricately linked with infections. Toll-like receptors (TLR) provide a potential link between infection and anti-myeloperoxidase (MPO) autoimmunity. TLR9 ligation has been shown to promote anti-MPO autoimmunity and glomerular vasculitis in murine MPO-AAV. This study investigates dendritic cell TLR9 ligation in murine experimental anti-MPO glomerulonephritis. We analyzed autoimmune responses to MPO following transfer of TLR9 stimulated, MPO pulsed dendritic cells and kidney injury following a sub-nephritogenic dose of sheep anti-mouse glomerular basement membrane globulin. TLR9 ligation enhanced dendritic cell activation upregulating CD40 and CD80 expression, promoting systemic anti-MPO autoimmunity and T cell recall responses and exacerbating kidney injury. CD40 upregulation by TLR9 was critical for the induction of nephritogenic autoimmunity. The presence of DEC205, which transports the TLR9 ligand to TLR9 located in the endosome, also promoted kidney injury. This confirms TLR9 mediated dendritic cell activation as a mechanism of anti-MPO autoimmunity in AAV and further defines the link between infection and the generation of MPO specific autoimmune inflammation.

## 1. Introduction

Anti-neutrophil cytoplasmic antibody (ANCA)-associated vasculitis (AAV) is a systemic autoimmune disease characterized by necrotizing inflammation of small to medium sized blood vessels. The most severe manifestations of AAV are rapidly progressive glomerulonephritis and pulmonary haemorrhage. Significant morbidity is associated with AAV and if untreated the mortality reaches 80% at 1 year [1] and up to 90% at 2 years [2].

The development of autoimmunity to the neutrophil protein myeloperoxidase (MPO) involves loss of tolerance to this self-antigen with activation of the innate immune system, production of MPO-ANCAs and the proliferation of effector anti-MPO CD4+ T cells [3]. Experimental and clinical evidence supports pathogenic roles for MPO-ANCAs and anti-MPO T cells in AAV but the origin of the anti-MPO autoimmune response is less well understood. Mechanisms proposed to induce the pathogenic anti-MPO autoimmune response in AAV include: exposure to exogenous antigens including infectious pathogens [4], exposure to endogenous auto-antigens such as peptides derived from alternative transcripts [5] or antisense peptides [6,7,8,9], exposure to auto-antigens generated following neutrophil degranulation forming extracellular traps (NETosis) [10,11,12], dysregulation in neutrophil apoptosis and clearance of apoptotic cells [13,14,15,16,17] and dysregulation of cell death machinery leading to necro-inflammatory auto-amplification loops [18]. An improved understanding of the critical molecular events that lead to the generation of anti-MPO autoimmunity in MPO-AAV will facilitate identification of specific targets for therapeutic intervention.

Whilst the pathogenesis of AAV is undoubtedly multifactorial with contributions from genetic factors and environmental exposure [19]; AAV is intricately linked with infections. Studies have documented seasonal variation in disease presentation suggesting correlations with microbial infection and infection precipitating disease onset and relapse [4]. Nasal colonization of *Staphylococcus aureus* is significantly increased in patients with granulomatosis with polyangiitis (GPA) and increases the relative risk of relapse over 7 fold [20]. Despite evidence linking infection with the development of AAV, few mechanistic links have been described.

Toll-like receptors (TLRs) are innate pattern recognition receptors, ubiquitously expressed on immune and resident tissue cells, whose ligation in response to bacterial and viral molecular patterns induces innate and subsequent adaptive immune responses. While TLRs are required for protection from invading microbes, inappropriate stimulation can result in the development of autoimmunity and organ injury [21] including kidney disease [22,23]. Pathogenic roles for TLRs have been demonstrated in experimental models of kidney disease including urinary tract infection [24,25], polyoma virus nephropathy [26], acute kidney injury [27], lupus nephritis [28] and glomerulonephritis [29,30,31].

TLR9, expressed intracellularly within the endosomal compartments of B lymphocytes, monocytes, neutrophils, natural killer cells, keratinocytes, melanocytes and dendritic cells, preferentially binds DNA motifs present on bacteria and viruses. TLR9 has been shown to be an important mediator in AAV: stimulation of peripheral blood mononuclear cells from GPA patients with a TLR9 ligand increased ANCA production [32], TLR9 expression is increased on B lymphocytes in patients with AAV; when these B lymphocytes are cultured with a TLR9 ligand they produce ANCAs [33] and when neutrophils are stimulated by TLR9 ligands MPO release and neutrophil degranulation is induced after Proteinase 3 (PR3)-ANCA stimulation [34].

TLR9 has been demonstrated to be an important mediator of autoimmunity and glomerular inflammation in a murine model of MPO-AAV. Summers et al. demonstrated TLR9 ligation promotes autoimmunity when administered to C57BL/6 mice in combination with murine MPO; glomerular vasculitis develops after the administration of a subnephritogenic dose of nephrotoxic serum to mice immunized with a TLR9 ligand and MPO [29]. The TLR9 ligand, CpG, has been demonstrated to bind to the cell surface receptor DEC205 resulting in the internalization of the CpG-DEC205 complex. CpG is then delivered to the surface of the endosome where it can associate with TLR9. DEC205 deficient mice demonstrate impaired dendritic and B cell maturation, impaired cytokine responses and suboptimal cytotoxic T cell responses [35]. The role of DEC205 as a cotransporter of CpG in the development of MPO-AAV is unknown.

Dendritic cells are key players in the development of autoimmunity as the antigen-presenting cell to naïve T cells, playing a critical role in the initiation and control of adaptive immune responses. Dendritic cells are activated by exogenous or endogenous ligands that bind PRRs, such as TLRs. Following TLR activation, dendritic cells upregulate costimulatory molecule expression and produce inflammatory cytokines that play crucial roles in T cell polarization.

The innate immune system’s discrimination of host from pathogen is not absolute, this being particularly true for the nucleic acid TLR agonists. As such, CpG DNA, the agonist of TLR9, is a chemical structure that is not unique to microbes. The ability to discriminate host nucleic acids from microbial nucleic acids appears to rely on a combination of properties of cells involved in the innate immune system. TLR recognition of host nucleic acids may be a promiscuity that has been tolerated throughout evolution, as the selective advantage of pathogen detection outweighs the risk of autoantibody production. We hypothesize that autoimmune recognition of MPO in MPO-AAV is facilitated by dendritic cell TLR9 ligation and sought to further examine the mechanism through which this occurs.

The present study utilized a murine model of MPO-AAV. Anti-MPO autoimmunity is induced and to trigger glomerular injury, a subsequent sub-nephritogenic (does not cause glomerular injury when injected into unsensitized wild type mice) dose of sheep anti-mouse glomerular basement membrane (GBM) globulin is used to recruit neutrophils to glomerular capillaries where they degranulate, depositing MPO and triggering anti-MPO CD4+ effector T cell responses [36]. The resultant histological injury, with focal and segmental necrosis in glomeruli, is characteristic of the vasculitic lesions seen in humans with MPO-AAV. Abnormal proteinuria is also seen.

Defining the molecular mechanisms of TLR9-induced anti-MPO autoimmunity and glomerular injury may identify critical therapeutic targets for patients with AAV.

## 2. Results

### 2.1. TLR9 Ligation Enhances MPO Pulsed Dendritic Cell Activation Upregulating Dendritic Cell CD40 and CD80 Expression

**Figure 1:**
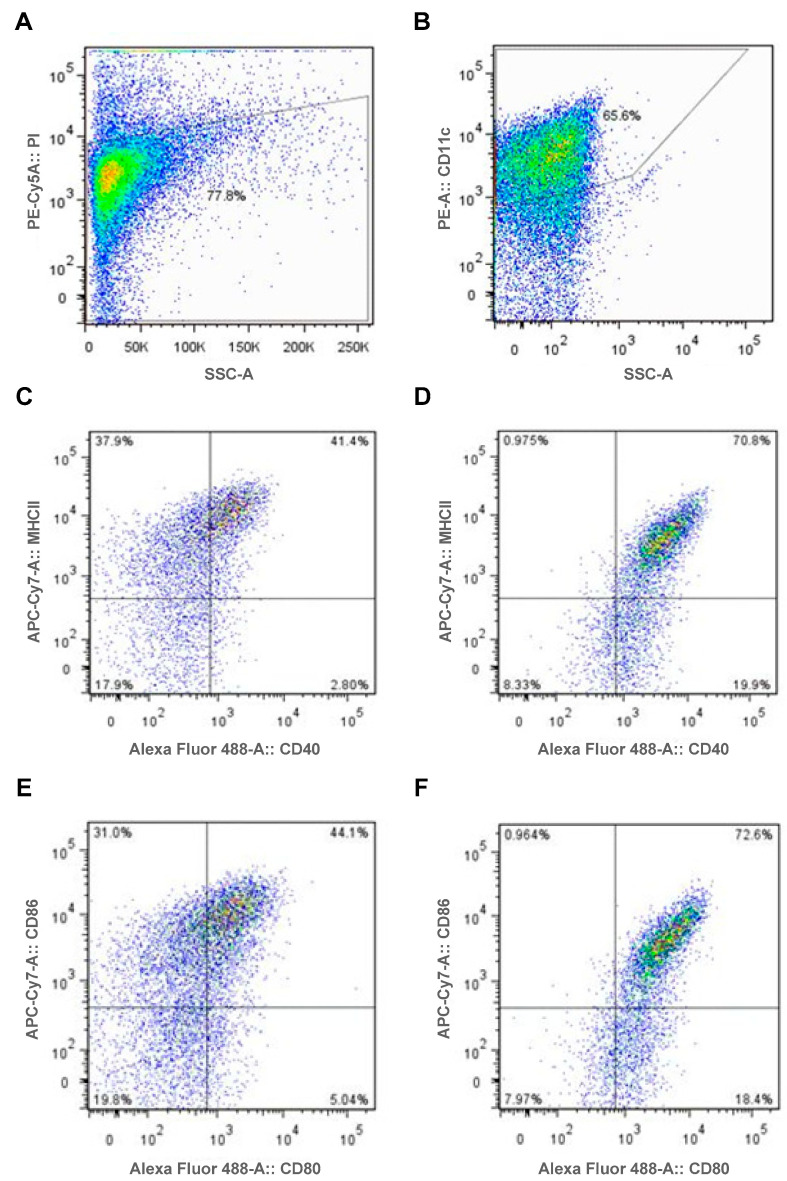
TLR9 ligation enhances MPO pulsed dendritic cell activation upregulating dendritic cell CD40 and CD80 expression. For fluorescence-activated cell sorting analysis, gates were set based on the mean fluorescence intensity for single color negative controls. (**A**,**B**): DCs assessed following rmMPO and TLR9 ligand, CpG incubation; Viability (**A**) and Cell Specificity (**B**). (**C**,**D**): DCs assessed following recombinant mouse MPO (rmMPO) and Control, GpC (**C**) and TLR9 ligand, CpG (**D**); Assessment of cell surface activation markers MHCII and CD40. CD40 expression was higher in rmMPO/CpG incubated DCs (91% CD40^hi^) compared to rmMPO/Control incubated DCs (44% CD40^hi^). MHCII expression was unchanged between DC groups ((**D**):72% vs. (**C**):79%). (**E**,**F**): DCs assessed following rmMPO and Control, GpC (**E**) and TLR9 ligand, CpG (**F**). Assessment of cell surface activation markers CD86 and CD80. CD80 expression was higher in rmMPO/CpG incubated DCs (91% CD80^hi^) compared to rmMPO/Control incubated DCs (39% CD80^hi^). CD86 expression was unchanged between DC groups ((**F**): 74% vs. (**E**): 75%). DC = Dendritic cell, PI = Propidium iodine.

Murine wild type (WT) dendritic cells were generated from bone marrow harvested from C57BL/6 mice. Dendritic cell viability and purity was assessed by flow cytometry. Dendritic cells were demonstrated to be 78% viable with viability assessed using Propidium iodine (PI, Figure 1A). The classical surface marker of dendritic cells CD11c was used to assess dendritic cell purity. Sixty-six percent of cells expressed CD11c (Figure 1B).

After 2 hours incubation with MPO and 6 hours stimulation with either the TLR9 ligand or control oligodendronucleotide (ODN) dendritic cells were assessed by flow cytometry for cell surface expression of MHCII, CD86, CD80 and CD40. Increased costimulatory molecule expression was seen in dendritic cells stimulated with the TLR9 ligand compared to control. Cell surface expression of CD40 increased in TLR9 stimulated cells when compared to controls (Figure 1C,D) as well as surface expression of CD80 (Figure 1E,F). MHCII (Figure 1C,D) and CD86 (Figure 1E,F) expression was similar between cells stimulated with the TLR9 ligand and control.

### 2.2. TLR9 Ligation of MPO Pulsed Dendritic Cells Promotes Systemic Anti-MPO Autoimmunity, T Cell Recall Responses and Subsequent Kidney Injury

**Figure 2:**
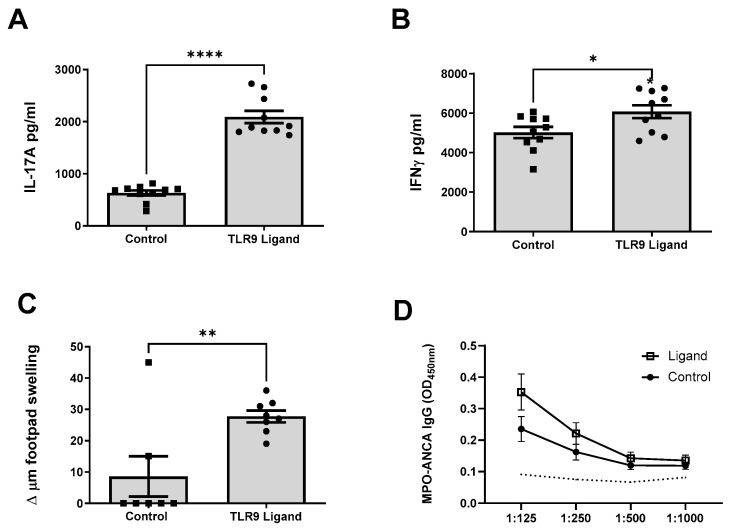
TLR9 ligation of MPO pulsed dendritic cells promotes early MPO autoimmunity. Early Immune Responses. MPO-stimulated mouse splenocyte cytokine production (day 10). (**A**): Increased production of interleukin 17A (IL-17A) in mice receiving TLR9 stimulated MPO pulsed DCs compared to Control stimulated MPO pulsed DCs. (**B**): Increased production of interferon gamma (IFN-γ) in mice receiving TLR9 stimulated MPO pulsed DCs. (**C**): Increased MPO specific dermal DTH response in mice receiving TLR9 stimulated MPO pulsed DCs. (**D**): No difference in serum MPO-ANCA was observed between groups. Dotted line represents the OD_450_ of sera from control, OVA immunized mice receiving anti-GBM globulin. Bars show the mean +/− SEM. * = *p* < 0.05, ** = *p* < 0.01, **** = *p* < 0.0001. DC = Dendritic cell. Mouse numbers for experiment: Control: n = 10, TLR9 Ligand: n = 10.

To determine if TLR9 stimulated MPO pulsed dendritic cells enhanced antigen specific splenocyte immune responses, signature Th17 and Th1 cytokines (IL-17A and IFN-γ, respectively), were assessed 10 days following dendritic cell immunization. Mice immunized with TLR9 stimulated MPO pulsed dendritic cells demonstrated a significant increase in MPO specific IL-17A and IFN-γ responses compared to mice immunized with control stimulated MPO pulsed cells (Figure 2A,B). The increased Th17 response was predominant in this the autoimmune phase of disease. After injecting MPO into the hind paw 24 h before mice were euthanized, only mice receiving TLR9 stimulated MPO pulsed dendritic cells developed detectable delayed type hypersensitivity (DTH) responses (Figure 2C). No difference in MPO-ANCA IgG titres were observed between groups (Figure 2D).

**Figure 3:**
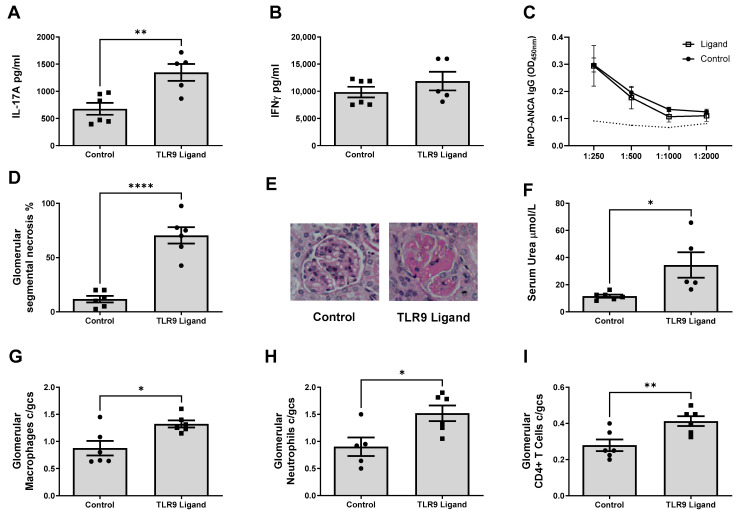
TLR9 ligation of MPO pulsed dendritic cells promotes late MPO autoimmunity, t cell recall responses and subsequent kidney injury. Immune responses (day 18): (**A**): Increased production of IL-17A in mice receiving TLR9 stimulated MPO pulsed DCs compared to control stimulated MPO pulsed DCs. (**B**): No significant increase in production of IFN-γ in mice receiving TLR9 stimulated MPO pulsed DCs at Day 18. (**C**): No difference in serum MPO-ANCA IgG in mice receiving TLR9 stimulated and control stimulated MPO pulsed DCs compared to OVA injected mice. Dotted line represents the OD_450_ of sera from control, OVA immunized mice receiving anti-GBM globulin. Glomerular Injury Measurements: (**D**,**E**): Kidney injury as assessed by percentage of abnormal glomeruli on formalin fixed paraffin embedded periodic acid Schiff sections taken at original magnification ×400. Increased kidney injury in mice receiving TLR9 stimulated MPO pulsed DCs. (**F**): Serum urea measurement in µmol/L. Increased in mice receiving TLR9 stimulated MPO pulsed DCs. (**G**): Increase in glomerular macrophage recruitment, measured as average FA11+ cells per glomerular cross section (cells/glom) in mice receiving TLR9 stimulated MPO pulsed DCs. (**H**): Increase in glomerular neutrophil recruitment, measured as average Gr1+ cells per glomerular cross section (cells/glom) in mice receiving TLR9 stimulated MPO pulsed DCs. (**I**): Increase in glomerular CD4+ T Cell recruitment, measured as average GK1.5+ cells per glomerular cross section (cells/glom) in mice receiving TLR9 stimulated MPO pulsed DCs. Bars show the mean +/− SEM. * = *p* <0.05, ** = *p* < 0.01, **** = *p* < 0.0001. DC = Dendritic cell. Mouse numbers for experiment: Control: n = 6, TLR9 Ligand: n = 6.

Th17 and Th1 immune responses were also assessed after triggering kidney injury (day 18); MPO stimulated splenocyte IL-17A production was enhanced in mice immunized with TLR9 stimulated MPO pulsed dendritic cells (Figure 3A). However, there was no significant difference in antigen stimulated splenocyte IFN-γ production following the kidney injury phase in mice receiving TLR9 stimulated dendritic cells compared to those receiving control stimulated cells (Figure 3B). Both control and TLR9 stimulated MPO pulsed dendritic cells developed MPO-ANCA IgG and there was no difference between groups. This was not observed in OVA immunized mice receiving anti-GBM globulin (OVA/FCA, OVA/FIA and anti-GBM, Figure 3C).

Having demonstrated enhanced anti-MPO autoimmunity after transfer of TLR9 stimulated MPO pulsed dendritic cells, we hypothesized that the enhanced immune responses would facilitate glomerular inflammation and injury. Mice were administered a sub-nephritogenic dose of sheep anti-mouse GBM globulin, which does not induce kidney injury if injected into naïve mice, but will only trigger glomerulonephritis in mice with established autoimmunity to MPO by recruiting neutrophils to the glomerulus where they degranulate, depositing MPO triggering anti-MPO T cell recall responses [36]. TLR9 stimulation of MPO pulsed dendritic cells led to an increase in histological kidney injury. More severe glomerular segmental necrosis was seen in mice receiving TLR9 stimulated dendritic cells than mice receiving control stimulated dendritic cells (Figure 3D,E). Mice immunized with TLR9 stimulated MPO pulsed dendritic cells also expectedly developed increased functional kidney injury with elevated serum urea compared to mice immunized with control stimulated MPO pulsed dendritic cells (Figure 3F).

Increased glomerular leukocyte recruitment occurred in mice immunized with TLR9 stimulated MPO pulsed dendritic cells demonstrating significantly increased macrophage (Figure 3G), neutrophil (Figure 3H), and CD4+ T cell (Figure 3I) glomerular recruitment.

To confirm the increased MPO autoimmunity and resultant kidney injury was TLR9 specific and reproducible, we utilized mice deficient in TLR9 (*Tlr9^−/−^*) to generate *Tlr9^−/−^* dendritic cells and compare to TLR9 stimulated MPO pulsed TLR9 intact C57BL/6 WT dendritic cells. Dendritic cells differentiated utilizing granulocyte-macrophage colony-stimulating factor (GM-CSF) from bone marrow harvested from *Tlr9^−/−^* and WT mice underwent 2 hour incubation with MPO followed by 6 hour stimulation with TLR9 ligand.

**Figure 4:**
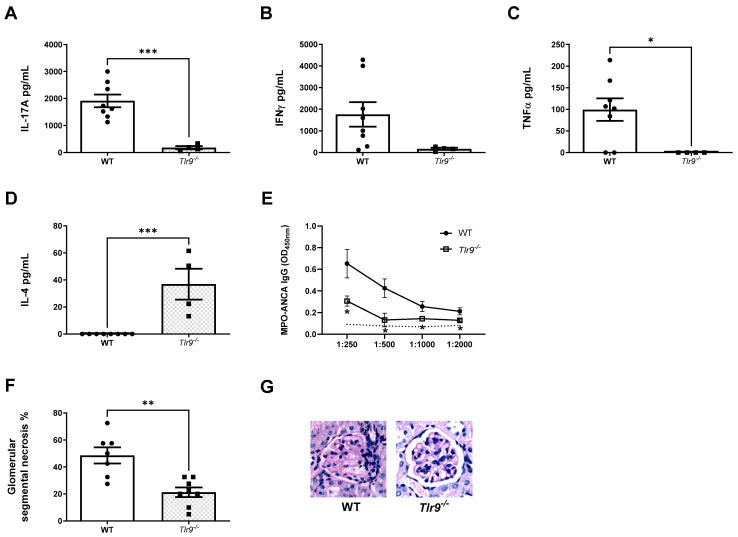
Enhanced TH17 Additionally, TH1 MPO autoimmunity induced by TLR9 stimulated MPO pulsed dendritic cells is dependent on TLR9 ligation. (**A**): Decreased production of IL-17A in mice receiving TLR9 stimulated MPO pulsed *Tlr9^−/−^* DCs compared to WT DCs. (**B**): Decreased (ns) production of IFN-γ in mice receiving TLR9 stimulated MPO pulsed *Tlr9^−/−^* DCs. (**C**): Decreased production of tissue necrosis factor alpha (TNFα) in mice receiving TLR9 stimulated MPO pulsed *Tlr9^−/−^* DCs. (**D**): Increased production of interleukin 4 (IL-4) in mice receiving TLR9 stimulated MPO pulsed *Tlr9^−/−^* DCs. (**E**): Decreased serum MPO-ANCA IgG in mice receiving TLR9 stimulated MPO pulsed *Tlr9^−/−^* DCs compared to WT DCs. Dotted line represents the OD_450_ of sera from control, OVA immunized mice receiving anti-GBM globulin. Kidney injury was assessed at Day 18. (**F**): Decreased kidney injury in mice receiving TLR9 stimulated MPO pulsed *Tlr9^−/−^* DCs. (**G**) Periodic-acid Schiff-stained micrographs of glomeruli were taken at original magnification ×400. Bars show the mean +/− SEM. * = *p* <0.05, ** = *p* < 0.01, *** = *p* < 0.001. DC = Dendritic cell. Mouse numbers for experiment: WT: n = 8, *Tlr9^−/−^*: n = 8.

Production of the signature Th17 cytokine, IL-17A, by MPO stimulated splenocytes was significantly attenuated in mice immunized with TLR9 stimulated MPO pulsed *Tlr9^−/−^* dendritic cells compared to those immunized with TLR9 stimulated MPO pulsed WT dendritic cells Figure 4A). A trend to decreased production of the dominant Th1 cytokine, IFN-γ, was also seen in mice receiving TLR9 stimulated MPO pulsed *Tlr9^−/−^* dendritic cells (Figure 4B). TNF-α production was abrogated in mice receiving TLR9 stimulated MPO pulsed *Tlr9^−/−^* dendritic cells compared to mice receiving WT cells (Figure 4C). Interestingly, mice receiving TLR9 stimulated MPO pulsed *Tlr9^−/−^* dendritic cells demonstrated increased antigen stimulated production of IL-4 compared to mice receiving WT cells (Figure 4D). Decreased serum MPO-ANCA IgG titres were seen in mice receiving TLR9 stimulated MPO pulsed *Tlr9^−/−^* dendritic cells compared to WT dendritic cells (Figure 4E). Kidney injury was ameliorated in mice receiving TLR9 stimulated MPO pulsed *Tlr9^−/−^* dendritic cells (Figure 4F,G).

### 2.3. CD40 Is a Critical Second Signal for the Induction of Nephritogenic Autoimmunity

Having demonstrated dendritic cell TLR9 ligation is required for the generation of systemic anti-MPO autoimmunity, anti-MPO T cell recall responses and subsequent kidney injury and further demonstrating that TLR9 ligation upregulates dendritic cell CD40 expression (Figure 1), the importance of CD40-induced dendritic cell activation as a second signal for the induction of nephritogenic autoimmunity was further assessed.

*CD40^−/−^* dendritic cells were generated; these cells were pulsed with MPO and stimulated with the TLR9 ligand and their ability to induce nephritogenic autoimmunity when transferred to naïve wild type mice was compared to TLR9 stimulated MPO pulsed WT dendritic cells.

Mice received 1 × 10^6^ TLR9 stimulated MPO pulsed *CD40^−/−^* dendritic cells or TLR9 stimulated MPO pulsed WT dendritic cells. Early developing anti-MPO immune responses were assessed at day 10 and both established immune responses and kidney injury were assessed following the induction of kidney injury as detailed previously.

**Figure 5:**
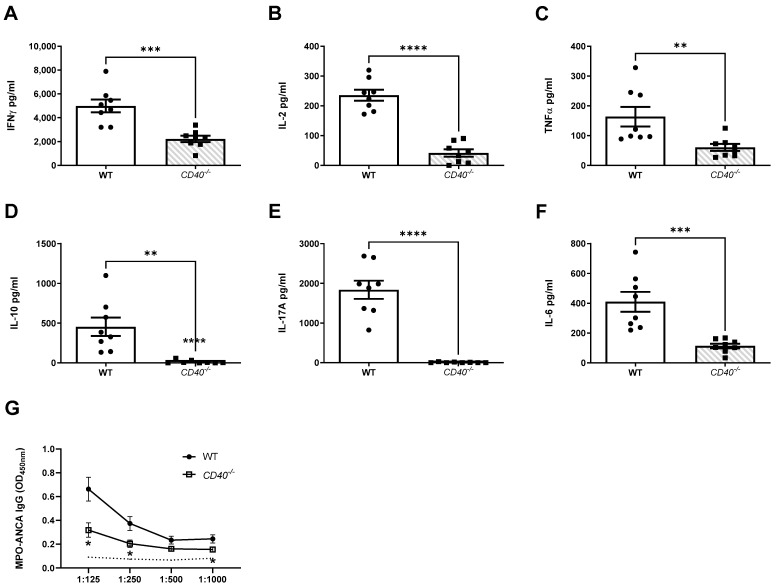
CD40 upregulation on TLR9 stimulated MPO pulsed dendritic cells is critical for the enhancement of MPO autoimmunity. Early Immune Responses Model (Day 10): (**A**): Reduced production of IFN-γ in mice receiving TLR9 stimulated MPO pulsed *CD40^−/−^* DCs compared to WT DCs. (**B**): Reduced production of interleukin 2 (IL-2) in mice receiving TLR9 stimulated MPO pulsed *CD40^−/−^* DCs. (**C**): Reduced production of TNFα in mice receiving TLR9 stimulated MPO pulsed *CD40^−/−^* DCs. (**D**): Reduced production of interleukin 10 (IL-10) in mice receiving TLR9 stimulated MPO pulsed *CD40^−/−^* DCs. (**E**): Reduced production of IL-17A in mice receiving TLR9 stimulated MPO pulsed *CD40^−/−^* DCs. (**F**): Reduced production of interleukin 6 (IL-6) in mice receiving TLR9 stimulated MPO pulsed *CD40^−/−^* DCs. (**G**): Decreased serum MPO-ANCA IgG in mice receiving TLR9 stimulated MPO pulsed *CD40^−/−^* DCs compared to WT DCs. Dotted line represents the OD_450_ of sera from control, OVA immunized mice receiving anti-GBM globulin. Bars show the mean +/− SEM. ** = *p* < 0.01, *** = *p* < 0.001, **** = *p* < 0.0001. DC = Dendritic cell. Mouse numbers for experiment: WT: n = 8, *CD40^−/−^*: n = 8.

Mice receiving TLR9 stimulated MPO pulsed *CD40^−/−^* dendritic cells had markedly reduced immune responses during the early autoimmune phase of disease. Th1, Th2 and Th17 immune responses were all significantly reduced. Th1 immune responses assessed included MPO specific splenocyte production of IFN-γ (Figure 5A), IL-2 (Figure 5B) and TNF-α (Figure 5C). Th2 immune response was assessed by production of IL-10 (Figure 5D). The Th17 response was assessed by production of IL-17A (Figure 5E) and IL-6 (Figure 5F). Consistently reduced Th17 immune responses were also seen following the induction of kidney injury. Humoral MPO-ANCA IgG responses were reduced in mice receiving TLR9 stimulated MPO pulsed *CD40^−/−^* dendritic cells compared to WT dendritic cells (Figure 5G).

**Figure 6:**
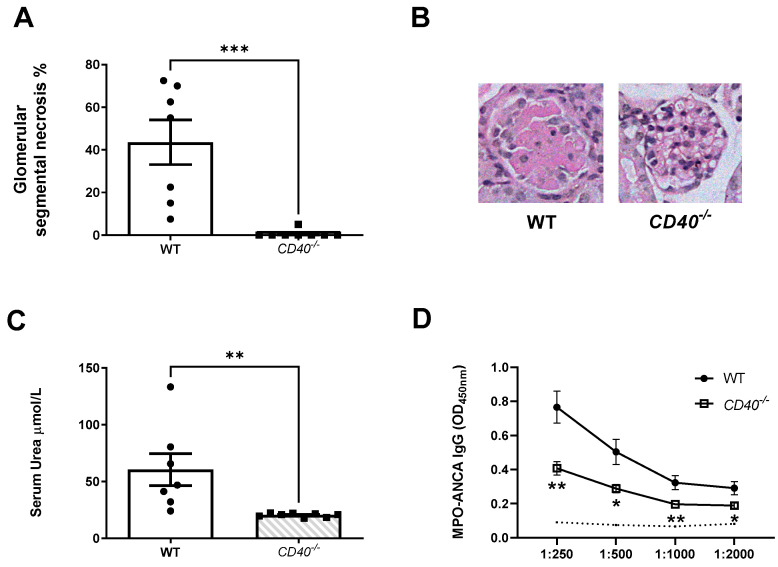
CD40 upregulation on TLR9 simulated MPO pulsed dendritic cells is critical for the development of kidney injury following the transfer of TLR9 stimulated MPO pulsed dendritic cells and the triggering of kidney injury. (**A**,**B**): Abrogated kidney injury in mice receiving TLR9 stimulated MPO pulsed *CD40^−/−^* DCs. Periodic-acid Schiff-stained micrographs of glomeruli were taken at original magnification ×400. (**C**): Serum urea measurement in µmol/L. Reduced in mice receiving TLR9 stimulated MPO pulsed *CD40^−/−^* DCs. (**D**): Decreased serum MPO-ANCA IgG in mice receiving TLR9 stimulated MPO pulsed *CD40^−/−^* DCs compared to WT DCs. Dotted line represents the OD_450_ of sera from control, OVA immunized mice receiving anti-GBM globulin. Bars show the mean +/− SEM. * = *p* < 0.05, ** = *p* < 0.01, *** = *p* < 0.001. DC = Dendritic cell. Mouse numbers for experiment: WT: n = 7, *CD40^−/−^*: n = 8.

Functional and histological kidney injury was ameliorated in keeping with overall reduced immune responses in mice receiving TLR9 simulated MPO pulsed *CD40^−/−^* dendritic cells. Mice receiving *CD40^−/−^* dendritic cells developed less severe glomerular segmental necrosis (Figure 6A,B) and had lower serum urea (Figure 6C). Decreased serum MPO-ANCA IgG titres were seen in mice receiving TLR9 stimulated MPO pulsed *CD40^−/−^* dendritic cells compared to WT dendritic cells following triggering of kidney injury (Figure 6D).

### 2.4. DEC205, a Cotransporter of the TLR9 Ligand, Facilitates the Development of Th17 Nephritogenic Autoimmunity and Kidney Injury

Having demonstrated TLR9 ligation of dendritic cells enhances MPO specific autoimmunity and subsequent kidney injury via increased dendritic cell activation mediated by expression of CD40, we further hypothesized that ligation of the cytoplasmic TLR9 receptor was facilitated by the dendritic cell surface receptor DEC205 which could represent an additional potential future therapeutic target for treatment of AAV.

*DEC205^−/−^* and WT dendritic cells were differentiated from bone marrow of *DEC205^−/−^* and WT mice, pulsed with MPO and stimulated with the TLR9 ligand and transferred into two groups of WT mice.

**Figure 7:**
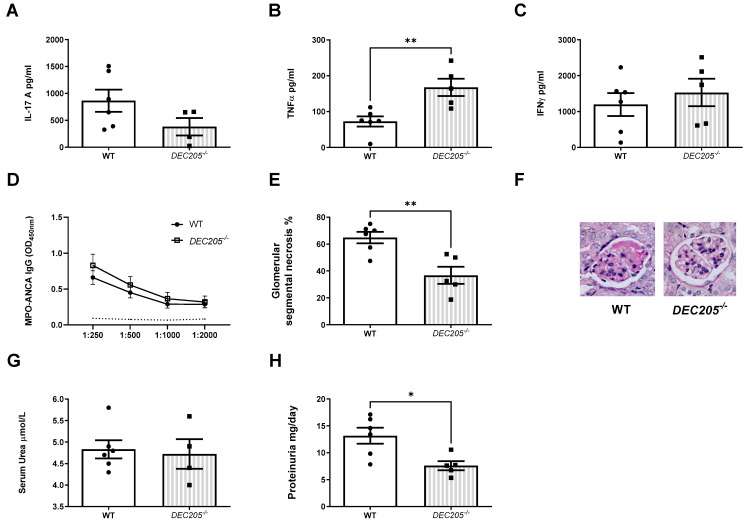
DEC205 is required for the development of Th17 MPO autoimmunity on transfer of TLR9 stimulated MPO pulsed dendritic cells. dendritic cell DEC205 deficiency protects from subsequent kidney injury. (**A**) Reduced production of IL-17A in mice receiving TLR9 stimulated MPO pulsed *DEC205^−/−^* DCs compared to WT DCs. (**B**): Increased production of TNF-α in mice receiving TLR9 stimulated MPO pulsed *DEC205^−/−^* DCs. (**C**): No difference in production of IFN-γ in mice receiving TLR9 stimulated MPO pulsed *DEC205^−/−^* DCs. (**D**): No difference in serum MPO-ANCA IgG in mice receiving both TLR9 stimulated MPO pulsed *DEC205^−/−^* and WT DCs compared to OVA injected mice. Dotted line represents the OD_450_ of sera from control, OVA immunized mice receiving anti-GBM globulin. (**E**,**F**): Decreased kidney injury in mice receiving TLR9 stimulated MPO pulsed *DEC205^−/−^* DCs. Periodic-acid Schiff-stained micrographs of glomeruli were taken at original magnification ×400. (**G**): Serum urea measurement in µmol/L. No difference in mice *DEC205^−/−^* TLR9 stimulated MPO pulsed *DEC205^−/−^* DCs. (**H**): Proteinuria measured in mg/day. Reduced in mice receiving TLR9 stimulated MPO pulsed *DEC205^−/−^* DCs. Bars show the mean +/− SEM. * = *p* <0.05, ** = *p* < 0.01. DC = Dendritic cell. Mouse numbers for experiment: WT: n = 6, *DEC205^−/−^*: n = 5.

An impaired Th17 immune response was seen with reduced MPO specific splenocyte IL-17A production in mice receiving TLR9 stimulated MPO pulsed *DEC205^−/−^* dendritic cells compared to those receiving WT dendritic cells (Figure 7A), although this did not reach statistical significance. Paradoxically, Th1 immune responses were enhanced with increased MPO specific splenocyte TNFα production following transfer of *DEC205^−/−^* dendritic cells (Figure 7B) and a trend towards increased MPO specific splenocyte IFN-γ production (Figure 7C). No differences in humoral responses (serum MPO-ANCA) were observed between groups (Figure 7D).

Kidney injury was attenuated in mice receiving *DEC205^−/−^* dendritic cells (Figure 7E,F). Whilst serum urea measurements did not differ between mice receiving *DEC205^−/−^* and WT cells (Figure 7F), 24 hourly protein excretion was reduced in those that received *DEC205^−/−^* dendritic cells reflecting less severe functional kidney injury (Figure 7G).

## 3. Discussion

In this study, we demonstrate that TLR9 ligation enhances MPO specific dendritic cell activation upregulating CD40 and CD80 expression. TLR9 stimulated MPO pulsed dendritic cells promote anti-MPO autoimmunity by driving Th1 and predominantly Th17 immune responses. Early studies demonstrated exaggerated Th1 immune responses in AAV patients, T cells from patients with active disease when stimulated exhibited a Th1 cytokine profile [37,38]. Subsequently, heightened Th17 immune responses have been demonstrated during active disease and remission [39,40]. Murine models of AAV have demonstrated important pathogenic roles for both Th1 and Th17 responses in the development of autoimmunity and kidney injury. Our study reflects the importance of both Th1 and Th17 responses in the pathogenesis of TLR9 mediated anti-MPO autoimmunity and consequent kidney injury. Our studies demonstrate a modest increase in Th1 responses in the autoimmune phase of TLR9-stimulated MPO-pulsed dendritic-cell-induced disease and a large and significant increase in Th17 responses in both the autoimmune and kidney injury phases. CD40 upregulation is shown to be a critical second signal to induce nephritogenic autoimmunity. The dendritic cell surface receptor DEC205 is shown to facilitate the ligation of TLR9, the increased Th17 immune response and the development of kidney injury. The enhanced MPO autoimmunity induced by TLR9 stimulated dendritic cells facilitates leukocyte recruitment into the glomerulus and histological and functional kidney injury akin to that seen in MPO-AAV.

Intracellular signals initiated by interaction of TLRs and PAMPs result in inflammatory responses, such as secretion of cytokines, type 1 interferon, chemokines and antimicrobial peptides. Maladaptation of such antimicrobial inflammatory responses has been proposed as the pathological initiating event for genetically susceptible patients. There is significant evidence identifying the linkage between infection and AAV [4]. Associations between infection and disease onset and relapse have been confirmed, in MPO-associated disease, respiratory infection has been shown to proceed severe crescentic glomerulonephritis [41,42,43]. Seasonal variation in AAV symptom onset has been observed. Correlation between nasal carriage of *Staphylococcus aureus* and disease relapse and reduced relapses with prophylactic antibiotic therapy both strongly point to the involvement of mechanisms associated with the immune response being implicated in the pathogenic process [20,44]. The inappropriate activation of Tolllike receptors involved in the anti-microbial immune response at a time of neutrophil activity increasing the local presence of MPO provides a potential pathophysiological linkage to explain the clinical links between infection and AAV.

The role of TLR9 in the induction and progression of autoimmunity is well appreciated [45]. TLR9 as a mediator of AAV has been demonstrated both clinically and experimentally. TLR9 ligands are able to trigger the production of ANCA by B lymphocytes from AAV patients in vitro [33]. Evidence of a strong association of TLR9 genotypes and haplotypes with GPA as well as a contrariwise association with microscopic polyangiitis was identified following genotyping of a large cohort of German AAV patients and further validated in Dutch and British cohorts [46].

We found that compared to mice receiving MPO pulsed dendritic cells stimulated with the control ODN, mice receiving MPO pulsed dendritic cells activated with a TLR9 ligand had modestly increased Th1 and profoundly increased Th17 anti-MPO immune responses and subsequent kidney injury. This data adds support to the hypothesis that infection, through TLR9 ligation, is a cofactor in the development of autoimmunity and kidney injury in AAV. This data further defines that dendritic cell TLR9 plays an important role in the development of autoimmunity in AAV and may represent a target for future therapeutic intervention.

The costimulatory molecule, CD40, is found on antigen presenting cells and is required for their activation. CD40 is essential in mediating a broad variety of immune and inflammatory responses including T cell-dependent immunoglobulin class switching, memory B cell development and germinal center formation [47]. CD40 has been implicated in the pathogenesis of numerous autoimmune diseases [48]. The soluble ligand of the CD40 receptor has been shown to have a strong association with disease activity in patients with active granulomatosis with polyangiitis [49]. We found CD40 to be upregulated on dendritic cells following exposure to the antigen MPO and activation with the TLR9 ligand. Dendritic cell CD40 upregulation following TLR9 ligand mediated activation was identified as a critical second signal for the induction of nephritogenic autoimmunity. Mice receiving TLR9 stimulated MPO pulsed CD40 deficient dendritic cells developed markedly reduced immune responses with near complete protection from kidney injury.

TLR9 is one of only four TLRs to be located intracellularly on the endosome therefore, TLR9 ligand utilizes a co-transporter, DEC205 to deliver CpG to the endosomal TLR9. We identified the presence of the dendritic cell surface receptor DEC205, a cotransporter of CpG to the intracellular TLR9 receptor, contributed to the development of Th17 immune responses and subsequent kidney injury in mice receiving TLR9 stimulated MPO pulsed dendritic cells. We also noted enhanced Th1 immune responses following transfer of TLR9 simulated, MPO pulsed DEC205 deficient dendritic cells. At this timepoint of this model after triggering kidney injury Th1 responses have been previously shown to predominate over Th17 responses which are most active in the early autoimmune phase of disease [50]. Here, we have shown evidence of increased Th17 and Th1 responses due to TLR9 ligation of dendritic cells with suppression of these responses when MPO pulsed, TLR9 stimulated *TLR9^−/−^* and *CD40^−/−^* dendritic cells were transferred to naïve mice. Recent evidence has suggested that conventional type 1 dendritic cells induce Th1, Th1-like follicular helper T cells and regulatory T cells after antigen boost via the DEC205 receptor [51]. This suggests that DEC205 may be involved in differential T cell lineage commitment. It is not known how DEC205 deficiency alters the Th1/Th2/Th17 balance nor if alternate cell surface receptors facilitate the delivery of ligands to the endosome when cell surface DEC205 is absent. Future investigation of the effects of DEC205 on T cell differentiation may yield valuable insights.

In summary this study demonstrates that MPO pulsed dendritic cells activated ex vivo through TLR9 ligation promote anti-MPO autoimmunity when transferred to naïve mice. Dendritic cell TLR9 ligation enhances activation upregulating cell surface CD40 expression. CD40 is a critical second signal for the induction of anti-MPO autoimmunity. The intracellular co-transporter, DEC205 is involved in the development of Th17 immune responses and kidney injury. This confirms TLR9 mediated dendritic cell activation as a mechanism of anti-MPO autoimmunity in AAV and further defines the link between infection and the generation of MPO specific autoimmune inflammation.

## 4. Materials and Methods

### 4.1. Mice

C57BL/6 (wild type), *Tlr9^−/−^* and *CD40^−/−^* male mice were bred at Monash Medical Centre Animal Facilities, Monash University, Australia. *DEC205^−/−^* male mice were obtained from Associate Professor Irina Caminschi (Department of Biochemistry and Molecular Biology, Biomedical Discovery Institute, Monash University). All mice were housed in specific pathogen free conditions at Monash Medical Centre, and studies were approved by Monash University Animal Ethics Committee in accordance with the Australian National Health and Medical Council animal experimentation guidelines. All experiments were undertaken when groups of mice were 7–10 weeks of age.

### 4.2. Reagents

Native murine MPO was purified from differentiated 32Dcl3 cells; an insect cell line (Sf21) was used to express recombinant mouse MPO as previously described [52]. The TLR9 ligand, CpG-containing oligonucleotide (CpG ODN) and TLR9 control, GpC containing oligonucleotide (GpC ODN), were manufactured by GeneWorks, Thebarton, Australia. The sequence for the immune-stimulatory CpG ODN was TCCATGACGTTCCTGACGTT (5′-3′) bound with phosphorothioate linkages. The sequence for the control GpC ODN was TCCATGAGCTTCCTGAGCTT (5′-3′) bound with phosphorothioate linkages. Recombinant mouse granulocyte macrophage colony stimulating factor (GM-CSF) was purchased from Gibco, Thermo Fisher Scientific, Waltham, MA, USA. Sheep anti-mouse GBM globulin was generated as previously described [53].

### 4.3. Experimental Design

Bone marrow derived dendritic cells were generated from bone marrow harvested from femurs of donor mice and cultured with GM-CSF for 8 days according to well established protocols [54]. Resultant dendritic cells were incubated at 37 °C, 5% CO_2_ seeded in supplemented RPMI culture medium (10% fetal calf serum (FCS), 2 mM L glutamine, 100 U/mL Penicillin Streptomycin and 50 μM 2-mercaptoethanol; Sigma Aldrich, Macquarie Park, Australia) at 1 × 10^6^ cells per mL for 2 h with 100 μg/mL recombinant mouse MPO and 10 ng/mL GM-CSF. Cells were then washed with RPMI/2.5% FCS and seeded in culture medium at 4 × 10^5^ cells per mL for 6 h incubation with 1 μg/mL TLR9 ligand or control. Cells were washed twice with RPMI/2.5% FCS and counted. Samples of cells from all cultures were analyzed with flow cytometry for viability, purity and cell surface marker expression. Cells were resuspended in normal saline; 1 × 10^6^ PI-, CD11c^hi^ cells in 200 μL saline were injected subcutaneously at the base of tail.

For experiments analyzing kidney injury, groups of mice were administered 1 × 10^6^ PI-, CD11c^hi^ dendritic cells subcutaneously at the base of tail. Seven days later 10 μg native mouse MPO in 100 μL of Freund’s Incomplete adjuvant (FIA; Sigma Aldrich, Macquarie Park, Australia) was administered via intraperitoneal injection. Glomerulonephritis was then triggered with 1.5 mg of sheep anti-mouse GBM globulin administered intravenously on day 14 and day 15 and kidney injury was assessed at day 18. C57BL/6 WT mice were immunized with control protein, ovalbumin (OVA) and GN induced by a sub-nephritogenic dose of sheep anti-mouse GBM globulin on day 14 and 15 as controls for experiments assessing MPO-ANCA IgG titres.

For experiments analyzing immune responses prior to the induction of glomerulonephritis, groups of mice were administered 1 × 10^6^ PI^-^, CD11c^hi^ dendritic cells subcutaneously at the base of tail. 10 days later immune responses were assessed.

MPO specific DTH responses were assessed by injecting 10μg of recombinant mouse MPO dissolved in 30 μL of phosphate-buffered saline (PBS) into the right hind paw and 30 μL of PBS into the left hind paw, 24 h before the termination of experiments. Differences in skin footpad thickness between right and left hind paws was quantified (Δμm) using a micrometer.

### 4.4. Fluorescence Activated Cell Sorting (FACS)

Dendritic cells were stained for 20 min with the following antibodies for analysis: FITC anti-CD40 (3/23), PE anti-CD11c (HL3) both from BD Biosciences; Franklin Lakes, NJ, USA, APC-Cy7 anti-MHCII (M5/114), APC-Cy7 antiCD86 both from BioLegend; San Diego, CA, USA and FITC anti-CD80 (16-10A1) from eBiosciences; Thermo Fisher Scientific, Waltham, MA, USA. Nonviable cells were identified by Propidium iodine (PI) staining. Cells were analyzed on the BD FACSCanto II Flow Cytometer machine; BD Biosciences, Franklin Lakes, NJ, USA using FACSDiva software; BD Biosciences, Franklin Lakes, NJ, USA.

### 4.5. Assessment of Systemic Autoimmune Response to MPO

For assessment of splenocyte cytokine production, a single cell suspension of mouse splenocytes was generated using aseptic technique. Cells were cultured at 4 × 10^6^ cells/mL per well in supplemented RPMI culture medium (10% fetal calf serum, 2 mM L glutamine, 100 U/mL Penicillin Streptomycin, 0.1 mg/mL streptomycin and 50μM 2-Mercaptoethanol; Sigma) with recombinant mouse MPO (10 μg/mL) at 37 °C, 5% CO_2_ for 72 h. IL-17A and IFN-γ concentrations were measured by either enzyme-linked immunosorbent assay (ELISA) or Cytometric cytokine bead array (BD Biosciences, Franklin Lakes, NJ, USA). For IFN-γ ELISAs the monoclonal antibodies used were rat anti-mouse IFN-γ (R4-6A2; BD Pharmingen, BD Biosciences, Franklin Lakes, NJ, USA) and biotinylated rat anti-mouse IFN-γ (XMG1.2; BD Pharmingen, BD Biosciences, Franklin Lakes, NJ, USA). IL-17A was measured using paired antibodies (DuoSet; R&D Systems, Minneapolis, MN, USA). IL-2, TNFα, IL-10, IL-4 and IL-6 concentrations were measured using Cytometric Bead Array Mouse Th1/Th2/Th17 cytokine kit from BD Biosciences, Franklin Lakes, NJ, USA as per manufacturer’s instructions.

For assessment of humoral anti-MPO responses ELISA was used to detect circulating serum anti-MPO IgG titers using a 96-well polystyerene microplate (Invitrogen Technologies, Thermo Fisher Scientific, Waltham, MA, USA). Plates were coated with 1 mg/mL rMPO, and serially diluted serum was added. Detection was by horseradish peroxidase–conjugated sheep anti-mouse IgG (1:2000; Amersham Biosciences, Rydalmere, Australia).

### 4.6. Assessment of Functional and Histological Kidney Injury

Histological assessment of kidney injury was assessed on 3 μm thick, formalin-fixed, paraffin-embedded, periodic acid-Schiff, stained kidney sections on coded slides. The percentage of glomeruli with segmental necrosis was determined by examining a minimum of 40 glomeruli per mouse for abnormalities. Serum urea measurements were performed by the Monash Health biochemistry department. Mice were housed in individual metabolic cages over the final 24 h of experiments to collect urine. Proteinuria was assessed using a Bradford’s assay and expressed as mg/24 h.

### 4.7. Immunohistochemical Analysis of Kidney Leukocyte Accumulation

Mouse kidney sections were fixed in periodate-lysine-paraformaldehyde for 4 h, washed with 20% sucrose solution and then mounted in OCT and frozen in liquid nitrogen. 6 μm tissue sections were cut and glomerular CD4+ T cells, macrophages and neutrophils were assessed by an immuno-peroxidase staining technique. The primary antibodies used were GK1.5 for CD4+ T cells (anti-mouse CD4; American Type Culture Collection, Manassas, VA, USA), FA/11 for macrophages (anti-mouse CD68 from Dr Gordon L. Koch, Cambridge, England) and RB6-8C5 for neutrophils (anti-Gr-1; DNAX, Palo Alto, CA, USA). A minimum of 30 consecutive glomeruli were assessed per mouse. Results were expressed as cells per glomerular cross-section.

### 4.8. Statistical Analysis

Data is expressed as mean +/− SEM. Groups of data were analyzed using Student’s *t*-test for analysis of 2 groups and one-way analysis of variance (Tukey’s post-test) for more than 2 groups. GraphPad Prism Software Inc., San Diego, CA, USA was used to analyze all data. *p* < 0.05 was considered statistically significant.

## Data Availability

All data is contained within the article. Not applicable.
